# Novel prognostic indicator combining inflammatory indicators and tumor markers for gastric cancer

**DOI:** 10.1186/s12957-023-02926-w

**Published:** 2023-02-18

**Authors:** Liang Yu, Runben Jiang, Wanjing Chen, Yanwei Liu, Gui Wang, Xin Gong, Yong Wang

**Affiliations:** The Second Hospital of Anhui Medical University, Hefei, 230601 Anhui China

**Keywords:** Gastric cancer, Inflammatory indicators, Tumor markers, Neutrophil-to-lymphocyte ratio, Carbohydrate antigen 19-9

## Abstract

**Background:**

Gastric cancer (GC) is one of the most common malignant tumors worldwide, and we hope to identify an economical but practical prognostic indicator. It has been reported that inflammatory indicators and tumor markers are associated with GC progression and are widely used to predict prognosis. However, existing prognostic models do not comprehensively analyze these predictors.

**Methods:**

This study retrospectively reviewed 893 consecutive patients who underwent curative gastrectomy from January 1, 2012, to December 31, 2015, in the Second Hospital of Anhui Medical University. Prognostic factors predicting overall survival (OS) were analyzed using univariate and multivariate Cox regression analyses. Nomograms including independent prognostic factors were plotted for predicting survival.

**Results:**

Ultimately, 425 patients were enrolled in this study. Multivariate analyses demonstrated that the neutrophil-to-lymphocyte ratio (NLR, total neutrophil count/lymphocyte count × 100%) and CA19-9 were independent prognostic factors for OS (*p*=0.001, *p*=0.016). The NLR-CA19-9 score (NCS) is constructed as the combination of the NLR and CA19-9. We defined NLR<2.46 and CA19-9≤37 U/ml as an NCS of 0, NLR≥2.46 or CA19-9>37 U/ml as an NCS 1, and NLR≥2.46 and CA19-9>37 U/ml as an NCS of 2. The results showed that higher NCS was significantly associated with worse clinicopathological characteristics and OS (*p*<0.05). Multivariate analyses revealed that the NCS was an independent prognostic factor for OS (NCS1: *p*<0.001, HR=3.172, 95% CI=2.120–4.745; NCS2: *p*<0.001, HR=3.052, 95% CI=1.928–4.832). Compared with traditional predictive indices, the NCS had the highest AUC for a 12-month survival, a 36-month survival, a 60-month survival, and OS (AUC= 0.654, 0.730, 0.811, 0.803, respectively). The nomogram had a higher Harrell’s C-index than the TNM stage alone (0.788 vs. 0.743).

**Conclusions:**

The NCS provides more accurate predictions of the prognosis of GC patients, and its predictive value is significantly better than that of traditional inflammatory indicators or tumor markers. It is an effective complement to existing GC assessment systems.

## Introduction

Gastric cancer (GC) is the fifth-most common malignancy in humans and ranks third in a tumor-related mortality according to the latest epidemiologic data [1]. Radical resection combined with chemotherapy has consistently been the core method for curing GC. Unfortunately, due to the highly aggressive nature of GC, almost 50% of patients suffer from tumor recurrence or metastasis after curative resection, and the 5-year survival rate remains less than 30% [2, 3]. Currently, the most common criteria used to predict GC patients’ long-term outcomes include the TNM staging system, tumor markers, and inflammation indicators, but clinical outcomes can vary in patients who have the same stages and similar treatment regimens [4–6], indicating that these systems provide incomplete prognostic information.

In 1863, Virchow first discovered the relationship between inflammation and cancer [7]; subsequently, an increasing number of related studies were carried out. A growing number of studies have proven the relationship between malignant tumors and inflammation [8–12]. Many scholars believe that immune status is closely related to survival in patients with various malignancies, including GC [6, 13–15]. The neutrophil-to-lymphocyte ratio (NLR) is a significant prognostic indicator of gastric cancer. Current researchers not only believe that gastric cancer patients with higher NLRs have a poorer prognosis [9, 16] but also indicate to a certain extent that the incidence of complications after surgery, such as anastomotic leakage, has increased [16, 17]. The NLR has been adopted for prognostic evaluation in many cancers, as well as the platelet-to-lymphocyte ratio (PLR) and lymphocyte-to-monocyte ratio (LMR).

The relationship between GC and tumor markers has basically been clarified, and a series of studies have explored the value of tumor markers in the diagnosis and prognosis of gastric cancer [18–20]. At the same time, tumor markers, such as carcinoembryonic antigen (CEA) and CA19-9, have been used to determine prognosis and monitor the therapeutic effects of treatments. The level of CEA may be increased in gastric carcinoma, lung carcinoma, and especially colorectal carcinoma, while CA19-9 is used mainly as a specific marker for pancreatic cancer.

However, these indicators remain controversial. For both inflammation indicators and tumor markers, the specificity and sensitivity in predicting the long-term outcome of GC patients alone are poor, and thus, there is an urgent need for a new, easy method to predict GC more accurately and perform targeted follow-up treatment and observation for patients who may have a poorer prognosis. Therefore, we attempted to combine inflammation indicators and tumor markers with a high predictive value to predict the prognosis of GC patients more accurately. In addition, we used prospective clinical data to investigate whether the new indicator could effectively predict postoperative outcomes of GC and compared its predictive value with other traditional indices.

## Materials and methods

### Patients

Between January 1, 2012, and December 31, 2015, a total of 893 consecutive patients admitted to the Second Hospital of Anhui Medical University were recruited for the trial. Patient eligibility criteria of this study included the following: (1) all patients who underwent gastrectomy with curative R0 resection; (2) postoperative pathology confirmed GC; (3) no active inflammatory, chronic infection, or autoimmune rheumatic diseases; and (4) no other malignancies. Patients who met the following criteria were excluded from this study: (1) serious complications or death that occurred within 15 days after operative, (2) treatment with neoadjuvant chemotherapy, (3) lack of inflammation and tumor marker data, and (4) acute complications such as perforation or bleeding. All patients, except for those with pTNM stage I, received 6–8 cycles of postoperative chemotherapy based on fluorouracil combined with platinum. This study was reviewed and approved by the Ethical Review Committee of the Second Hospital of Anhui Medical University, and the approved number is YX2021-138(f1). The patients were informed orally or in writing about the relevant matters of the study. All patients expressed their complete understanding of the study and signed an informed consent form.

### Data collection

The collection of clinical indicators included basic demographic information (age, sex), routine blood tests (total peripheral neutrophils, lymphocyte count, monocyte count, platelet count, CEA, CA19-9), and tumor-related information (size, depth, differentiation, Borrmann type, lymph node, distant metastasis, pathological stage). All blood test data were collected 1 week before surgery. The NLR was calculated by dividing the neutrophil count by the lymphocyte count. The PLR was calculated by dividing the platelet count by the lymphocyte count. The LMR was calculated by dividing the lymphocyte count by the monocyte count. The clinical stage of GC was determined following the eighth American Joint Committee on Cancer (AJCC) guidelines. The optimal cutoff values of CEA (5 ng/ml) and CA19-9 (37 U/ml) were determined by the standards of our hospital.

### Follow-up

After surgery, all patients were followed by radiology and laboratory tests every 3 months for 2 years and every 6 months for 2–5 years. In addition, examinations, including chest radiography, endoscopy, or abdominal and pelvic CT, were performed at least once per year. The follow-up period lasted 5 years after surgery or to the date of death. The overall survival (OS) was defined as the time interval from surgery to the last follow-up or to death from any cause.

### Statistical analysis

Data are presented as mean ± SD for normally distributed data and median (interquartile range) for data not-normally distributed. Receiver operating characteristic (ROC) curves were generated. The optimal cutoff values of the NLR, PLR, and LMR were obtained through the ROC curves by calculating the Youden indices corresponding to different cutoff values of each inflammatory index in the ROC curve, and the corresponding cutoff values of the maximum value of the Youden index were used to divide the patients into two groups. A Cox proportional hazards regression model was used to identify the independent predictors associated with OS, and variables with a value of *p*< 0.05 in the univariate analysis were subsequently included in a multivariate analysis. OS was assessed using the Kaplan-Meier method. Differences between the areas under the curve (AUCs) of each inflammatory and tumor marker were compared to determine the predictive value of each index for OS and the postoperative setting time points.

Models with independent prognostic factors were selected to plot the nomogram. The predictive values for survival were determined by Harrell’s C-index. In theory, the higher the C-index is the more precise the prognosis prediction. The decision curve analysis (DCA) plot can be used to represent the model with the greatest net benefits that had the highest clinical use, and it has been widely used to estimate whether the clinical use of diagnostic tests and prediction models would do more good than harm [21]. In the current study, DCA was conducted to evaluate the clinical use of the nomogram by quantifying the net benefits over the AJCC staging system. The calibration curve, the best method to visually compare the accordance between the predicted risk and the actual absolute risk, was used to evaluate the consistency of the model. If the calibration curve of the prediction model was closer to the standard curve, the consistency of the nomogram was better [22].

All statistical analyses were performed using SPSS v.22.0 for Windows (SPSS Inc., Chicago, IL, USA). The nomogram, DCA, and calibration curves were plotted with R Studio (version 1.1.463, with packages “rms,” “survival,” “hmisc,” and “rmda”). *p* values less than 0.05 were considered statistically significant.

## Results

### Patient clinicopathological characteristics

**Fig. 1:**
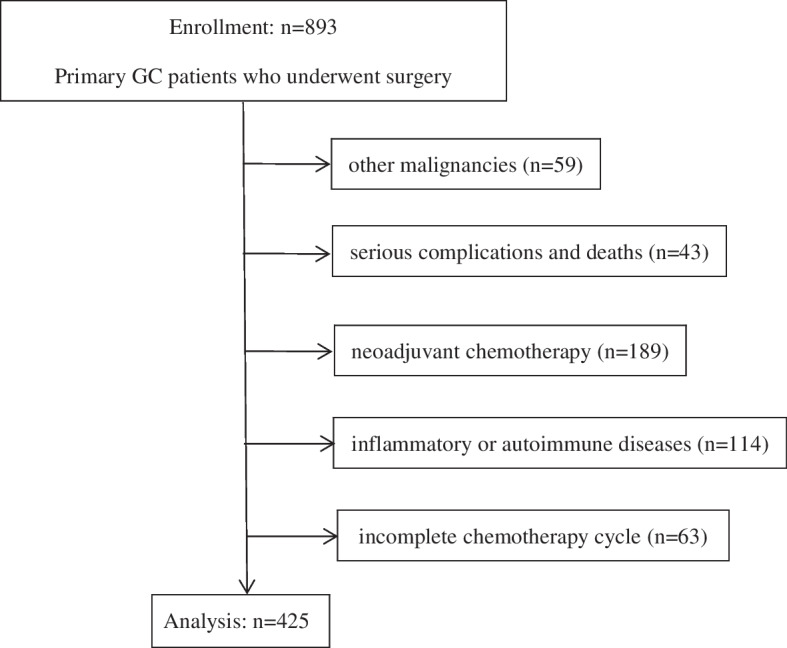
A total of 893 patients underwent gastrectomy for GC at the Second Hospital of Anhui Medical University between January 1, 2012, and December 31, 2015. Finally, a total of 425 patients were selected for the study. GC gastric cancer

Overall, 425 patients were included in the study (Fig. 1). There were 311 (73.18%) males and 114 (26.82%) females. According to the eighth edition of the AJCC staging system, a total of 68 patients (16.00%) had TNM stage I, 162 patients (38.12%) had TNM stage II, 163 patients (38.35%) had TNM stage III, and 32 patients (7.53%) had TNM stage IV. All patients in TNM stage IV were GC patients with isolated hepatic metastases. According to the standard of surgical treatment of gastric cancer, we performed radical resection of the primary and metastatic lesions in these patients. Regarding Borrmann types, 230 patients (54.12%) had Borrmann type I-II, and 195 patients (45.88%) had Borrmann type III-IV. In addition, there were 168 patients (39.53%) with tumor sizes greater than 5 cm and 184 patients (43.29%) with poor differentiation. The characteristics are detailed in Table 1.

**Table 1:** Patients clinicopathological characteristics

Variables	*n* (%) or median (IQR)
Sex	
Female	114 (26.82%)
Male	311 (73.18%)
Age	
<65years	216 (50.82%)
≥65years	209 (49.18%)
Tumor size	
<5cm	257 (60.47%)
≥5cm	168 (39.53%)
Differentiation	
Moderate/well	241 (56.71%)
Poor	184 (43.29%)
Borrmann type	
I–II	230 (54.12%)
III–IV	195 (45.88%)
Tumor depth	
T1	45 (10.59%)
T2	47 (11.06%)
T3	230 (54.12%)
T4	103 (24.24%)
Lymph node	
N0	100 (23.53%)
N1	181 (42.59%)
N2	103 (24.24%)
N3	41 (9.65%)
Distant metastasis	
M0	393 (92.47%)
M1	32 (7.53%)
pTNM stage	
I	68 (16.00%)
II	162 (38.12%)
III	163 (38.35%)
IV	32 (7.53%)
CEA	
≤5ng/ml	276 (64.94%)
>5ng/ml	149 (35.06%)
CA19-9	
≤37U/ml	317 (74.59%)
>37U/ml	108 (25.41%)
NLR	2.71 (1.73–4.46)
PLR	136.8 (94.0–207.6)
LMR	4.46 (2.87–6.27)

*NLR* the neutrophil-to-lymphocyte ratio, *PLR* the platelet-to-lymphocyte ratio, *LMR* the lymphocyte-to-monocyte ratio

### Survival analysis

**Fig. 2:**
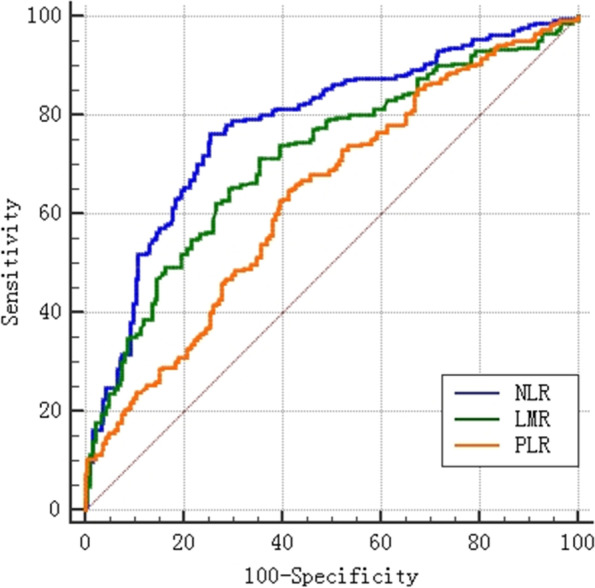
The optimal cutoff points of preoperative NLR, PLR, and LMR for postoperative survivals were obtained by calculating the maximum Youden index of the ROC curves. NLR the neutrophil-to-lymphocyte ratio, PLR the platelet-to-lymphocyte ratio, LMR the lymphocyte-to-monocyte ratio

**Fig. 3:**
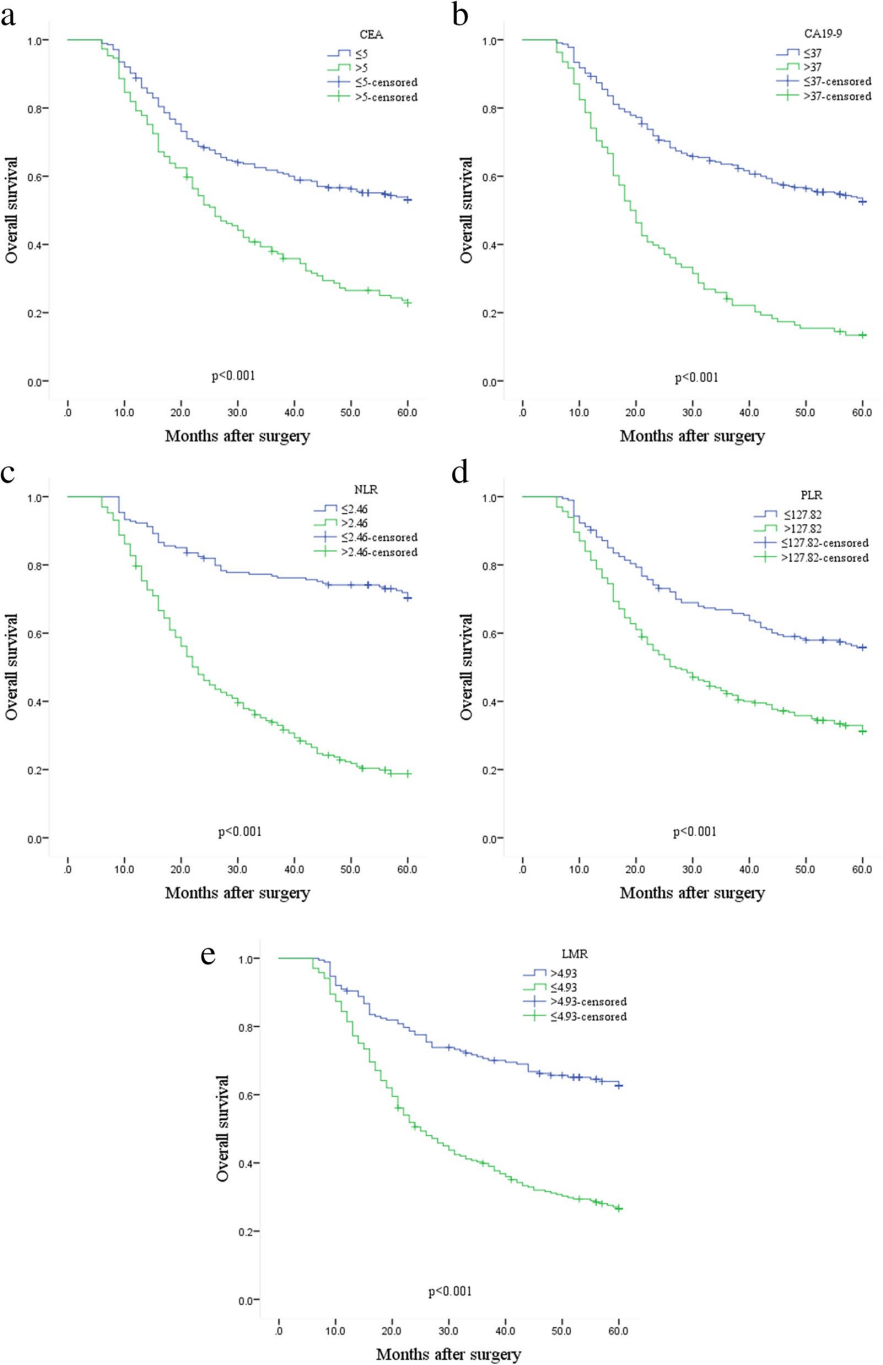
Kaplan-Meier estimates of the OS for patients according to CEA (**a**), CA19-9 (**b**), NLR (**c**), PLR (d), and LMR (**e**). NLR the neutrophil-to-lymphocyte ratio, PLR the platelet-to-lymphocyte ratio, LMR the lymphocyte-to-monocyte ratio. OS overall survival

In this study, 24 patients were lost during follow-up, with 12-, 36-, and 60-month OS rates of 87.3%, 53.9%, and 43.4%, respectively. The median survival time was 42 months. The optimal cutoff points for preoperative NLR, PLR, and LMR for postoperative survivals were obtained by calculating the maximum Youden index of the ROC curves, which were 2.46, 127.8, and 4.93, respectively (Fig. 2, Table 2), and based on these values, the entire sample was divided into two groups to identify the independent predictors associated with survival. The results of univariate analysis for the whole sample revealed that age, tumor size, differentiation, Borrmann type, tumor depth, lymph node involvement, distant metastasis, pTNM, CEA, CA19-9, NLR, PLR, and LMR were related to OS. Further multivariate analyses revealed that age, tumor depth, pTNM, CA19-9, and NLR were independent risk factors for OS (*p*=0.038, 0.009, <0.001, 0.016, 0.001, respectively) (Table 3). Kaplan–Meier survival curves comparing the OS of each hematological parameter showed that elevated CEA, CA19-9, NLR, and PLR and decreased LMR were associated with reduced OS (Fig. 3a–e).

**Table 2:** The optimal cutoff points of NLR, PLR, and LMR

	Cutoff value	AUC (95%CI)	maximal Youden index	*p*
NLR	2.46	0.778 (0.734–0.823)	0.508	<0.001
PLR	127.8	0.635 (0.582–0.688)	0.234	<0.001
LMR	4.93	0.287 (0.238–0.336)	0.361	<0.001

*NLR* the neutrophil-to-lymphocyte ratio, *PLR* the platelet-to-lymphocyte ratio, *LMR* the lymphocyte-to-monocyte ratio

**Table 3:** Univariate and multivariate analyses of variables associated with OS

Variables	Univariate analysis		Multivariate analysis	
	Hazard ratio (95%CI)	*p*	Hazard ratio (95%CI)	*p*
Sex		0.728		
Female	1			
Male	1.052 (0.790–1.403)			
Age		<0.001		0.038
<65 years	1		1	
≥65 years	1.963 (1.516–2.541)		1.342 (1.017–1.771)	
Tumor size		<0.001		
<5cm	1			
≥5cm	1.666 (1.294–2.147)			
Differentiation		0.008		
Moderate/well	1			
Poor	1.409 (1.094–1.814)			
Borrmann type		<0.001		
I–II	1			
III–IV	1.614 (1.253–2.079)			
Tumor depth		<0.001		0.009
T1	1		1	
T2	4.305 (1.215–15.258)	0.024	1.218 (0.202–7.341)	0.829
T3	11.662 (3.710–36.658)	<0.001	1.200 (0.176–8.203)	0.852
T4	41.409 (13.073–131.166)	<0.001	2.228 (0.317–15.649)	0.421
Lymph node		<0.001		
N0	1			
N1	5.937 (3.398–10.375)	<0.001		
N2	10.133 (5.736–17.902)	<0.001		
N3	15.816 (8.540–29.291)	<0.001		
Distant metastasis		<0.001		
M0	1			
M1	4.458 (3.022–6.577)			
pTNM stage		<0.001		<0.001
I	1			
II	6.519 (2.623–16.200)	<0.001	2.673 (0.501–14.263)	0.250
III	25.415 (10.389–62.172)	<0.001	5.570 (0.915–33.897)	0.062
IV	51.590 (19.885–133.844)	<0.001	11.700 (1.921–71.263)	0.008
CEA		<0.001		
≤5ng/ml	1			
>5ng/ml	2.098 (1.626–2.706)			
CA19-9		<0.001		0.016
≤37U/ml	1		1	
>37U/ml	2.937 (2.256–3.823)		1.442 (1.072–1.939)	
NLR		<0.001		0.001
≤2.46	1		1	
>2.46	4.468 (3.303–6.045)		1.772 (1.245–2.522)	
PLR		<0.001		
≤127.8	1			
>127.8	1.979 (1.518–2.580)			
LMR		<0.001		
≤4.93	1			
>4.93	0.365 (0.276–0.484)			

(1) *NLR* the neutrophil-to-lymphocyte ratio, *PLR* the platelet-to-lymphocyte ratio, *LMR* the lymphocyte-to-monocyte ratio. (2) *OS* overall survival

### Novel prognosis score factor

**Fig. 4:**
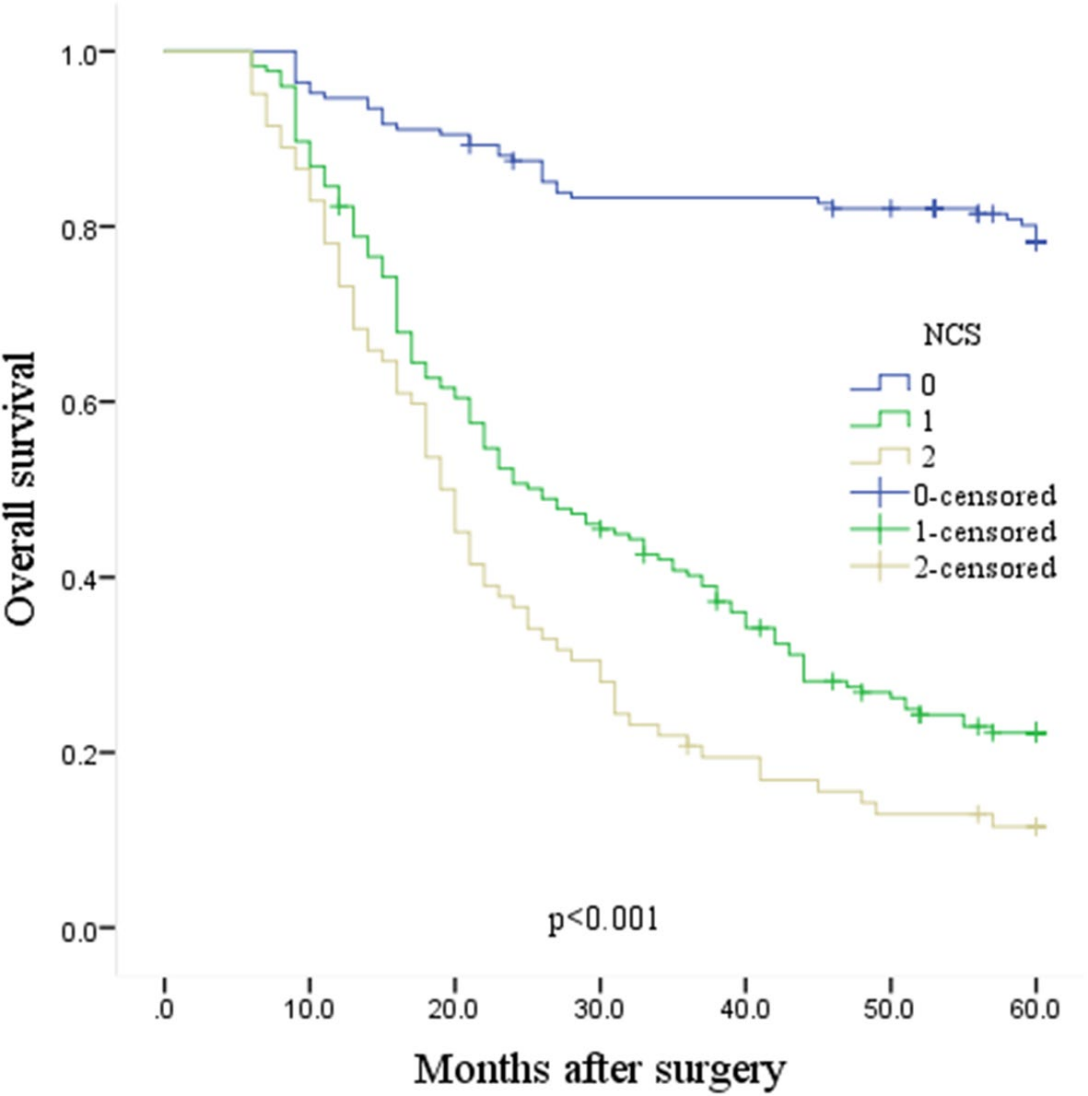
Kaplan-Meier estimates of the OS for patients according to NCS. NCS the NLR-CA19-9 score. OS overall survival

According to the results of multivariate analysis, a novel prognostic prediction system involving the NLR-CA19-9 score (NCS), which combines the CA19-9 level and the NLR, was established. Based on this, the scoring standard of the comprehensive index was obtained, which ranged from 0 to 2. We defined NLR<2.46 and CA19-9≤37 U/ml as an NCS of 0, NLR≥2.46 or CA19-9>37 U/ml as an NCS 1, and NLR≥2.46 and CA19-9>37 U/ml as an NCS of 2. The NCS was 0 for 168 (39.53%) patients, 1 for 175 (41.18%) patients, and 2 for 82 (19.29%) patients. The association between the NCS and the clinicopathological characteristics of patients with GC is demonstrated in Table 4. A higher NCS was significantly associated with worse clinicopathological characteristics, such as tumor depth, lymph node involvement, pTNM stage, CEA, PLR, and LMR. We continued to analyze the statistical relationship of the NCS and other clinicopathological characteristics with survival. The results showed that the NCS was also an independent prognostic factor for postoperative OS in GC patients, in addition to age, tumor depth, and pTNM (*p*<0.001, 0.024, 0.018, and <0.001, respectively) (Table 5). Similarly, we performed Kaplan-Meier survival curves based on the NCS, and the results showed that the higher the NCS was, the shorter the survival period, which indicated a worse prognosis (Fig. 4).

**Table 4:** Statistical relationship between the NCS and other variables

Variables	NCS			*p*
	0 *n* (%)	1 *n* (%)	2 *n* (%)	
Sex				0.585
Female	44 (26.19%)	51 (29.14%)	19 (23.17%)	
Male	124 (73.81%)	124 (70.86%)	63 (76.83%)	
Age				<0.001
<65years	106 (63.10%)	85 (48.57%)	25 (30.49%)	
≥65years	62 (36.90%)	90 (51.43%)	57 (69.51%)	
Tumor size				0.001
<5cm	120 (71.43%)	97 (55.43%)	40 (48.78%)	
≥5cm	48 (28.57%)	78 (44.57%)	42 (51.22%)	
Differentiation				0.252
Moderate/well	100 (9.52%)	101 (57.71%)	40 (48.78%)	
Poor	68 (40.48%)	74 (42.29%)	42 (51.22%)	
Borrmann type				0.001
I–II	109 (64.88%)	87 (49.71%)	34 (41.46%)	
III–IV	59 (35.12%)	88 (50.29%)	48 (58.54%)	
Tumor depth				<0.001
T1	35 (20.83%)	10 (5.71%)	0 (0)	
T2	30 (17.86%)	15 (8.57%)	2 (2.44%)	
T3	90 (53.57%)	100 (57.14%)	40 (48.78%)	
T4	13 (7.74%)	50 (28.57%)	40 (48.78%)	
Lymph node				<0.001
N0	70 (41.67%)	24 (13.71%)	6 (7.32%)	
N1	63 (37.50%)	81 (46.29%)	37 (45.12%)	
N2	30 (17.86%)	48 (27.43%)	25 (30.49%)	
N3	5 (2.98%)	22 (12.57%)	14 (17.07%)	
Distant metastasis				<0.001
M0	166 (98.81%)	157 (89.71%)	70 (85.37%)	
M1	2 (1.19%)	18 (10.29%)	12 (14.63%)	
pTNM stage				<0.001
I	53 (31.55%)	14 (8.00%)	1 (1.22%)	
II	75 (44.64%)	69 (39.43%)	18 (21.95%)	
III	38 (22.62%)	74 (42.29%)	51 (62.20%)	
IV	2 (1.19%)	18 (10.29%)	12 (14.63%)	
CEA				<0.001
≤5ng/ml	130 (77.38%)	117 (66.86%)	29 (35.37%)	
>5ng/ml	38 (22.62%)	58 (33.14%)	53 (64.63%)	
PLR				<0.001
≤127.8	112 (66.67%)	62 (35.43%)	20 (24.39%)	
>127.8	56 (33.33%)	113 (64.57%)	62 (75.61%)	
LMR				<0.001
≤4.93	52 (30.95%)	114 (65.14%)	71 (86.59%)	
>4.93	116 (69.05%)	61 (34.86%)	11 (13.41%)	

(1) *PLR* the platelet-to-lymphocyte ratio, *LMR* the lymphocyte-to-monocyte ratio. (2) *NCS* the NLR-CA19-9 score

**Table 5:** Univariate and multivariate analyses incorporating the NCS

Variables	Univariate analysis		Multivariate analysis	
	Hazard ratio (95%CI)	*p*	Hazard ratio (95%CI)	*p*
Sex		0.728		
Female	1			
Male	1.052 (0.790–1.403)			
Age		<0.001		0.024
<65years	1		1	
≥65years	1.963 (1.516–2.541)		1.375 (1.042–1.813)	
Tumor size		<0.001		
<5cm	1			
≥5cm	1.666 (1.294–2.147)			
Differentiation		0.008		
Moderate/well	1			
Poor	1.409 (1.094–1.814)			
Borrmann type		<0.001		
I–II	1			
III–IV	1.614 (1.253–2.079)			
Tumor depth		<0.001		0.018
T1	1		1	
T2	4.305 (1.215–15.258)	0.024	1.332 (0.221–8.038)	0.755
T3	11.662 (3.710–36.658)	<0.001	1.240 (0.181–8.488)	0.827
T4	41.409 (13.073–131.166)	<0.001	2.202 (0.313–15.492)	0.428
Lymph node		<0.001		
N0	1			
N1	5.937 (3.398–10.375)	<0.001		
N2	10.133 (5.736–17.902)	<0.001		
N3	15.816 (8.540–29.291)	<0.001		
Distant metastasis		<0.001		
M0	1			
M1	4.458 (3.022–6.577)			
pTNM stage		<0.001		<0.001
I	1		1	
II	6.519 (2.623–16.200)	<0.001	2.396 (0.446–12.859)	0.308
III	25.415 (10.389–62.172)	<0.001	5.445 (0.889–33.357)	0.067
IV	51.590 (19.885–133.844)	<0.001	10.274 (1.677–62.953)	0.012
CEA		<0.001		
≤5ng/ml	1			
>5ng/ml	2.098 (1.626–2.706)			
PLR		<0.001		
≤127.8	1			
>127.8	1.979 (1.518–2.580)			
LMR		<0.001		
≤4.93	1			
>4.93	0.365 (0.276–0.484)			
NCS		<0.001		<0.001
0	1		1	
1	5.874 (4.048–8.524)	<0.001	3.172 (2.120–4.745)	<0.001
2	8.710 (5.795–13.093)	<0.001	3.052 (1.928–4.832)	<0.001

(1) *PLR* the platelet-to-lymphocyte ratio, *LMR* the lymphocyte-to-monocyte ratio. (2) *NCS* the NLR-CA19-9 score

### Predictive value

**Fig. 5:**
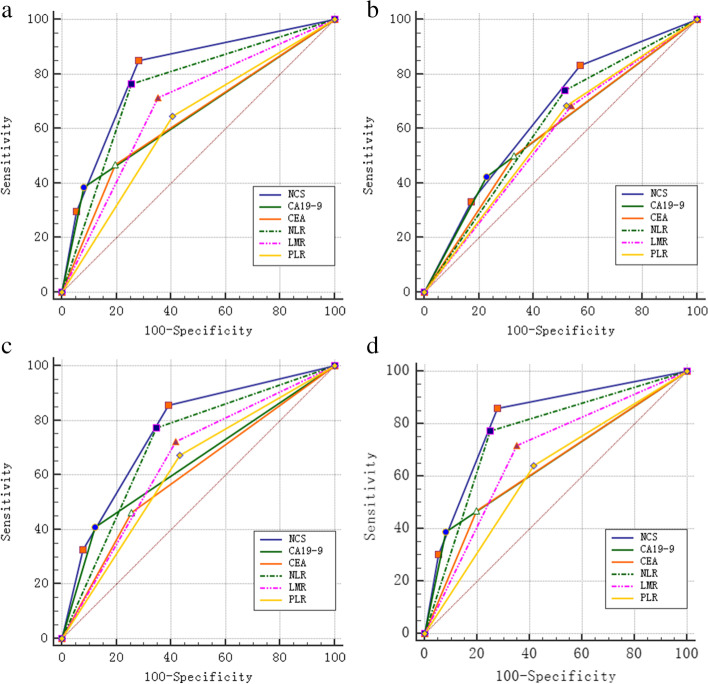
ROC analysis of NCS, CA19-9, CEA, NLR, PLR, LMR for OS, 1-, 3-, and 5-year survival (**a**–**d**) after the operation. NCS the NLR-CA19-9 score, NLR the neutrophil-to-lymphocyte ratio, PLR the platelet-to-lymphocyte ratio, LMR the lymphocyte-to-monocyte ratio, OS overall survival

To compare the predictive power of the NCS with other hematological parameters for OS and 12-, 36-, and 60-month survival, we compared the AUC of each inflammatory and tumor marker with that of the NCS. It was confirmed that the NCS had the highest AUC (0.803, 0.763, 0.811, respectively) for OS and 36- and 60-month survival, respectively, and the differences were all statistically significant (*p*<0.05) (Fig. 5a–d, Table 6). The higher AUC further confirmed the favorable sensitivity and specificity of the NCS.

**Table 6:** Comparison of the AUC between inflammatory indicators and tumor markers

Variables	OS			12 months			36 months			60mo		
	AUC (95%CI)	*z* statistic	p*	AUC (95%CI)	*z* statistic	p*	AUC (95%CI)	*Z* statistic	p*	AUC (95%CI)	z statistic	p*
CEA	0.637 (0.589–0.682)	5.962	<0.001	0.586 (0.537–0.633)	1.768	0.077	0.603 (0.554–0.649)	5.734	<0.001	0.636 (0.589–0.682)	6.270	<0.001
CA19-9	0.652 (0.605–0.697)	8.562	<0.001	0.598 (0.550–0.645)	2.138	0.033	0.644 (0.597–0.690)	6.726	<0.001	0.655 (0.608–0.700)	8.841	<0.001
NLR	0.754 (0.710–0.794)	4.039	<0.001	0.613 (0.565–0.660)	1.707	0.088	0.713 (0.668–0.756)	3.676	<0.001	0.761 (0.717–0.801)	4.089	<0.001
PLR	0.620 (0.572–0.666)	6.818	<0.001	0.581 (0.533–0.629)	1.651	0.099	0.619 (0.570–0.665)	5.311	<0.001	0.613 (0.565–0.659)	7.419	<0.001
LMR	0.680 (0.634–0.724)	4.711	<0.001	0.573 (0.534–0.621)	2.385	0.017	0.653 (0.605–0.698)	4.406	<0.001	0.683 (0.636–0.727)	4.936	<0.001
NCS	0.803 (0.762–0.840)	-	-	0.654 (0.607–0.699)	-	-	0.763 (0.720–0.803)	-	-	0.811 (0.770–0.847)	-	-

(1) *NLR* the neutrophil-to-lymphocyte ratio, *PLR* the platelet-to-lymphocyte ratio, *LMR* the lymphocyte-to-monocyte ratio. (2) *NCS* the NLR-CA19-9 score

*Comparison of AUC between the NLR-CA19-9 score and other variables

### Nomogram

**Fig. 6:**
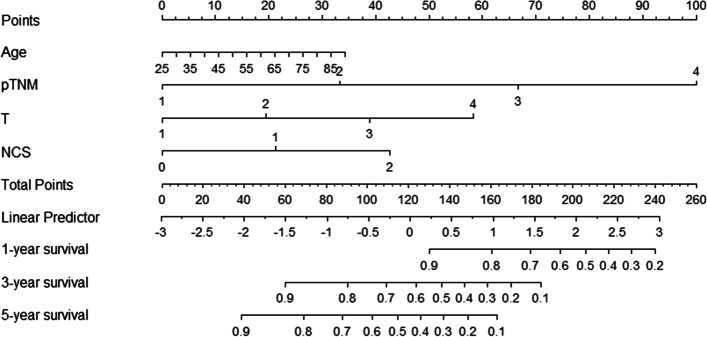
Nomogram to predict 1-, 3-, and 5-year OS for GC patients. NCS the NLR-CA19-9 score, OS overall survival

To make individualized predictions of the survival probability in all GC patients, we combined all independent prognostic factors, including age, TNM stage, tumor depth, and NCS, as described in detail in Table 4, and established a nomogram for the entire cohort (Fig. 6).

**Fig. 7:**
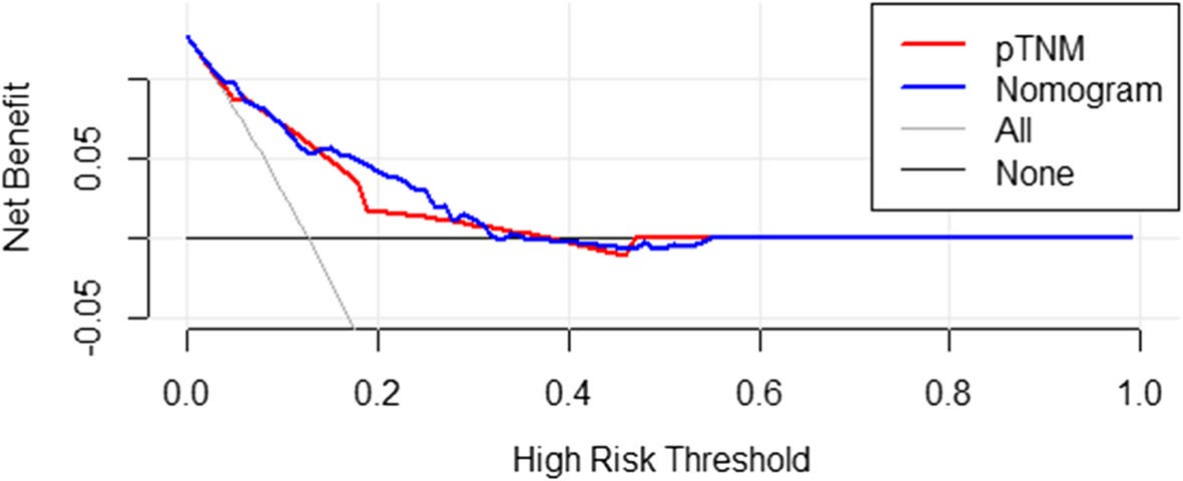
The DCA of the nomogram and the AJCC TNM staging system to OS. AJCC American Joint Committee on Cancer, OS overall survival

**Fig. 8:**
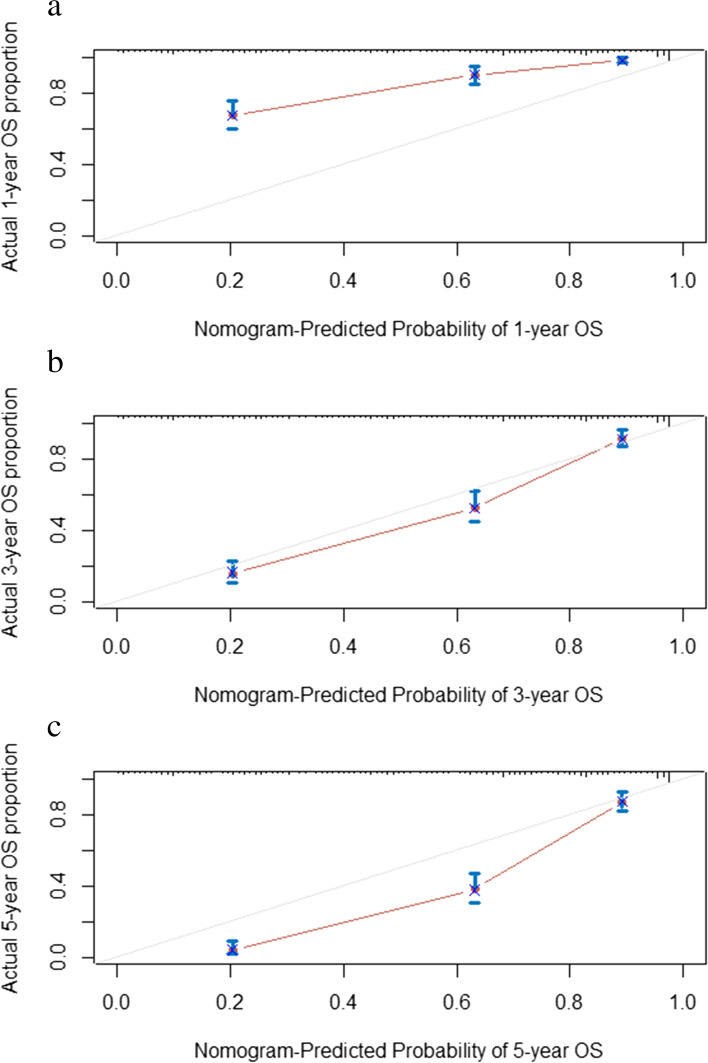
The calibration curves to predict 1-, 3-, and 5-year OS (**a**–**c**). OS overall survival

Discrimination and calibration are both important features of model performance. In this study, Harrell’s C-index of our nomogram (0.788, 95% CI 0.813–0.762) was higher than that of the TNM stage alone (0.743, 95% CI 0.770–0.717), indicating a more accurate and robust performance estimate. In addition, the DCA of the novel nomogram revealed a superior net clinical benefit over the 8th AJCC TNM staging system alone and exhibited higher clinical use than the AJCC staging system in OS prediction (Fig. 7). Furthermore, the calibration plots showed super agreement between the actual and predicted survival (Fig. 8a–c). All the above results show the better predictive capability of the established nomogram over the existing AJCC staging system.

## Discussion

Advances in modern medicine have allowed scholars to gradually turned their attention to the early treatment and follow-up of various malignant tumors, which puts forward extremely high requirements on how to distinguish patients with a poor prognosis. To date, researchers have established several scoring systems that reflect inflammation or tumor status [23–25]. Our study enrolled 425 GC patients and found that the NLR and CA19-9 were independent prognostic factors for postoperative OS. Based on this, we developed a novel index combining these inflammatory and tumor markers and confirmed that the new index could provide better prognostic value than either the NLR or CA19-9 alone.

Inflammation is an important characteristic of the tumor microenvironment and is associated with the promotion, progression, and metastasis of tumors [8]. Tumor cells produce cancer-related inflammatory mediators, resulting in relative neutrophilia, thrombocytosis, and lymphocytopenia. These phenomena cause an elevated NLR and PLR [26] and further affect the occurrence, progression, and metastasis of tumors [27]. Neutrophils are currently believed to promote cancer cell proliferation and metastasis by producing proangiogenic chemokines and vascular endothelial growth factor [28–30], and lymphocytes are antitumor factors involved in cytotoxic activity [31]. Numerous studies have proven the relationship between a high NLR and poor outcomes in various malignancies, such as gastric cancer [9, 16], colon cancer [13], and pancreatic cancer [32]. The present study revealed the NLR as an independent factor with a cutoff value of 2.46, and its AUC was higher than that of other hematological parameters.

Carbohydrate antigen (CA) 19-9 is one of the most common tumor markers of gastric cancer, and positivity is frequently related to tumor stage, poor prognosis, recurrence, and metastasis [18–20]. Jing et al. [19] found that surgery can significantly reduce the level of CA19-9; if the level returns to normal after surgery, the prognosis is not significantly different from that of patients with normal CA19-9 before surgery. As confirmed in our study, the prognosis of GC patients with low levels of CA19-9 before surgery was significantly better than that of patients with high levels. Of course, we consider the NLR and CA19-9 to represent not only simple changes in several indicators but also the balance of tumor and antitumor status in the body. When this balance is broken, tumor promotion is prioritized, leading to a poor prognosis.

Unfortunately, previous conclusions on these indicators have not always been consistent. Some scholars have insisted that there was no significant correlation between the preoperative NLR and survival time in GC patients [33–35]. In addition, controversy about CA19-9 also exists. Researchers have reported a low positive rate of CA19-9 in GC patients [36, 37]. In addition, some scholars believe that CA19-9 cannot accurately predict the prognosis of patients with GC [18], especially early GC [37]. This means that using an indicator alone to predict prognosis may lose some potential information, resulting in limited predictive value. In this study, we discussed the predictive significance of various inflammatory indicators and tumor markers in the prognosis of GC in detail and innovatively introduced a new index, the NLR-CA19-9 score (NCS). We found that the NCS not only could serve as an independent prognostic factor but also had a higher AUC than other inflammatory and tumor markers for OS, 12-, 36-, and 60-month survival, which means that the NCS has a stable prognostic ability that was better than that of other indicators. It was worth noting that there were no significant differences in AUC between the NCS and NLR, PLR, or CEA at 12 months after surgery. We believed that the survival of patients at 12 months after surgery was relatively higher (87.3%), resulting in no statistically significant difference. With the progress of follow-up, the survival of patients had declined, and the differences had gradually become apparent. Therefore, the differences were all statistically significant for OS and 36- and 60-month survival (*p*<0.05). Moreover, a model including the NCS (C-index, 0.788) was superior to that constructed without it (C-index, 0.743) in predicting OS after radical gastrectomy. Importantly, the novel index in our model could be conveniently attained from routine laboratory inspection, which was advantageous because it avoids the potential unavailability of other predictors.

The results of this study that the NCS, as a novel scoring system, has favorable sensitivity and specificity in predicting the long-term postoperative outcomes of GC patients and could provide more precise and informative prognostic value than other indicators. This new indicator may have vital use in predicting recurrence, therapeutic intervention, and surveillance strategies, especially when incorporating the TNM stage. For example, patients with a higher NCS could be strongly recommended to receive postoperative multimodal treatment, such as chemotherapy, immunotherapy, or targeted therapy and should receive a more rigorous follow-up schedule, with comprehensive medical examinations at least every 3 months for 5 years, such as gastrointestinal endoscopy, CT with contrast, and serum tumor markers, which may provide a survival benefit for GC patients.

Several limitations remain in our present study. First, the main drawback of our study is its retrospective nature, and bias in the process of patient selection cannot be avoided. Second, because the specific time of tumor recurrence in some patients cannot be completely determined, we did not study the relationship between various indicators and postoperative progression-free survival (PFS). Third, the pathological stages of the patients in our study are relatively broad, so the conclusion may not apply to patients with a particular TNM stage. But we are still expanding the sample size to verify whether our conclusion are applicable to similar stages.

## Conclusion

In conclusion, to the best of our knowledge, the NCS is an independent prognostic factor for OS in GC patients and provides great value in predicting postoperative overall survival. It is an effective complement to existing GC assessment systems. Future studies should consider combining the NCS into the current TNM system to more specifically predict the prognosis of GC patients, who will be likely to benefit from a rigorous follow-up strategy.

## Data Availability

The datasets generated and/or analyzed during the current study are not publicly available due to personal request but are available from the corresponding author on reasonable request.
